# Network pharmacognosy of *Galphimia glauca*: Mapping the molecular landscape of a traditional Mexican medicinal plant

**DOI:** 10.1371/journal.pone.0317546

**Published:** 2025-07-01

**Authors:** María José Cambero Acosta, Guillermo de Anda-Jáuregui

**Affiliations:** 1 Computational Genomics Department, Instituto Nacional de Medicina Genómica, Ciudad de México, México; 2 PREGEP, Colegio de Postgraduados, Ciudad de México, México; 3 Researchers for Mexico program, CONAHCYT, Ciudad de México, México; 4 Center for Complexity Sciences, UNAM, Ciudad de México, México; Baotou Medical College, CHINA

## Abstract

This study explores the pharmacological landscape of *Galphimia glauca*, a traditional Mexican medicinal plant known for its sedative and anti-inflammatory effects. Using network pharmacognosy, we analyzed the interactions of *G. glauca*’s bioactive compounds, Galphimines A-I, with human protein targets. SwissTargetPrediction identified 214 unique protein targets across the galphimines, revealing a core-periphery structure in a bipartite network where 41 targets are shared among all compounds. Further interaction analysis using STRING-DB generated a dense protein-protein interaction network comprising 1,386 connections. Centrality analysis highlighted proteins such as SRC, MTOR, and MAPK3 as key nodes involved in cell growth, proliferation, and immune regulation pathways, suggesting these as pivotal mediators of *G. glauca*’s pharmacological effects. Community detection with the Walktrap algorithm further segmented the network into functionally relevant modules linked to cell survival, immune response, and inflammation, reflecting the therapeutic effects historically attributed to *G. glauca*. Our findings underscore the plant’s multi-target therapeutic potential and highlight the value of network-based approaches in understanding traditional medicine. This work lays the groundwork for further studies aimed at refining therapeutic strategies based on *G. glauca*’s bioactive compounds and suggests network pharmacognosy as a promising tool for assessing other traditional medicinal plants.

## Introduction

*Galphimia glauca* is a widely used plant in traditional Mexican medicine, recognized for its therapeutic sedative and tranquilizing effects [1]. This shrub, commonly referred to as red arnica, belongs to the Malpighiaceae family and grows between one and three meters in height. Characterized by clusters of yellow flowers, *G. glauca* has ovate or elongated leaves that are green on top and bluish underneath. It is a native species in Mexico, thriving in mountainous regions as well as in flatlands across central and northeastern states such as Aguascalientes, Guanajuato, and Jalisco, where it grows wild [2].

The bioactive compounds of *G. glauca*, known as Galphimines A-I, are responsible for its various medicinal properties, including antioxidant, antiproliferative, proapoptotic, antiangiogenic, and anti-inflammatory activities [3]. The antioxidant properties help inhibit oxidative processes, reducing free radical production. Its antiproliferative effects suppress the growth of cells, particularly cancer cells, through pathways like JAK (Janus kinase 2) and STAT (signal transducer and activator of transcription). Proapoptotic effects induce programmed cell death, helping eliminate damaged cells. The antiangiogenic activity inhibits the formation of new blood vessels, primarily through interactions with tyrosine kinases, while anti-inflammatory effects modulate immune responses, addressing chronic inflammation linked to diseases such as arthritis and neurodegenerative disorders.

In recent years, understanding the complex interactions of medicinal compounds has become increasingly important in developing safe and effective treatments. *Network pharmacognosy* applies network science to the pharmacology of natural products, examining how multiple compounds in a medicinal plant, like *G. glauca*, interact with various molecular targets and pathways. This approach helps visualize the interconnected effects of *G. glauca*’s compounds on human health, providing insights into its medicinal applications and guiding the development of more precise therapeutic agents without adverse side effects.

In this work, we aim to characterize the pharmacological landscape of *Galphimia glauca* by mapping the predicted interactions of its major bioactive compounds with human protein targets. By applying a network pharmacognosy approach, we seek to identify shared and unique molecular targets, uncover functional modules, and provide mechanistic insight into the plant’s reported therapeutic effects.

## Results and discussion

### Identification of pharmacological targets

The bioactive compounds of *Galphimia glauca*, known as Galphimines (A-I), were analyzed using the SwissTargetPrediction tool to identify their potential pharmacological targets. The results identified a total of 180 distinct protein targets across all galphimines, with varying probabilities of interaction. A comprehensive set of selected interactions for all targets can be found in S1 File.

### Target space of galphimines

**Fig 1:**
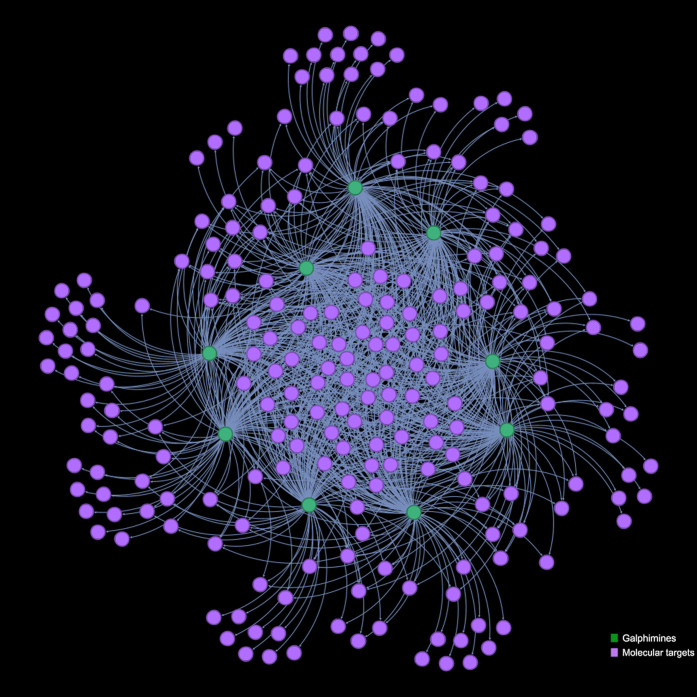
Network visualization of the target space of *G. glauca,* represented as a bipartite network. Galphimines are shown in green, and molecular targets in purple. A core-periphery structure may be observed in the network. A high-resolution version of this image is provided in S2 File.

Analysis of the target space for *Galphimia glauca* revealed a single, connected network component composed of 214 proteins associated with Galphimines A–I. Within this set, 41 targets were shared across all compounds, suggesting convergence on molecules linked to common biological processes. The remaining targets were compound-specific, pointing to additional functional diversity. A visualization of this network is shown in Fig 1.

**Fig 2:**
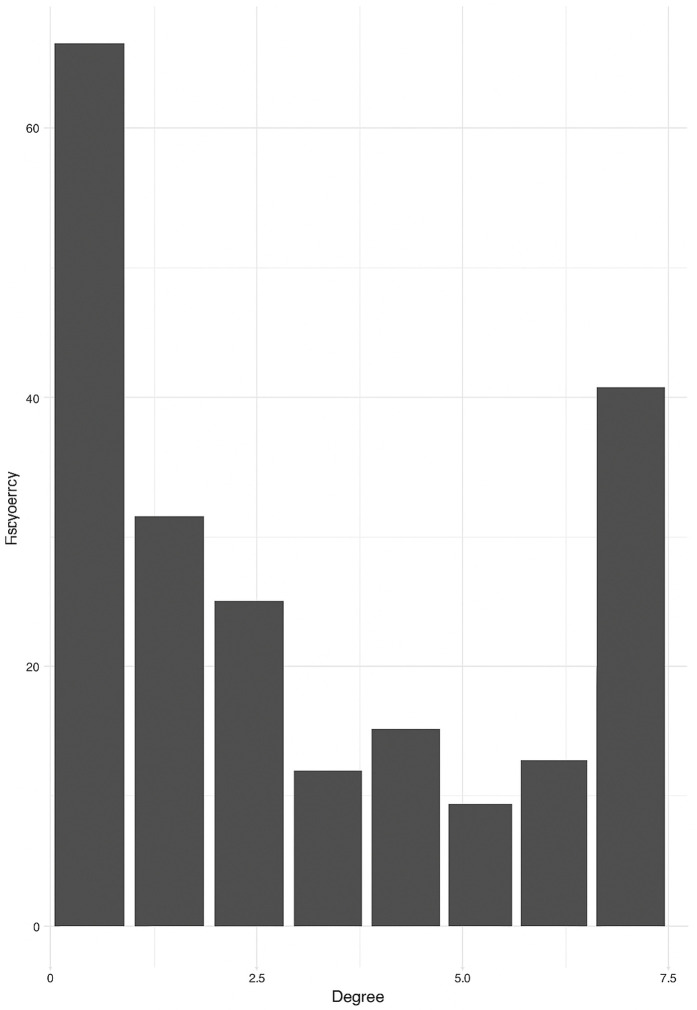
Degree distribution of target proteins in the *G. glauca* target space. Notice that the core structure (nodes connected to all galphimines) and the periphery structure (nodes connected to a single galphimine) are more abundant in the target space.

We represented this target space as a bipartite network anchored by a **core structure** of 41 shared targets surrounded by a **peripheral layer** of 63 unique targets, each associated with a single galphimine. The degree distribution of these targets, shown in Fig 2, reflects their connectivity within the network. Full descriptors and data for all targets are available in S2 File.

Biologically, several targets within the core structure point to possible pharmacological activities of *G. glauca*. Noteworthy proteins such as Protein Kinase C Alpha (PRKCA), Glucocorticoid Receptor (NR3C1), and P-Glycoprotein 1 (ABCB1) were identified across multiple compounds, suggesting that these shared targets could contribute to the therapeutic effects reported for *G. glauca*.

The bipartite structure of the galphimine–target network reveals a pharmacological architecture that combines a highly shared core of protein targets with a diverse periphery of compound-specific interactions. This configuration suggests that the therapeutic effects of *G. glauca* may be mediated by molecules associated with broadly relevant mechanisms, such as inflammation and stress response, as well as by more specific pathways linked to individual galphimines. These findings highlight the importance of considering multi-compound, multi-target interactions when analyzing traditional medicinal plants.

### Protein interaction network of *G. glauca* targets

**Fig 3:**
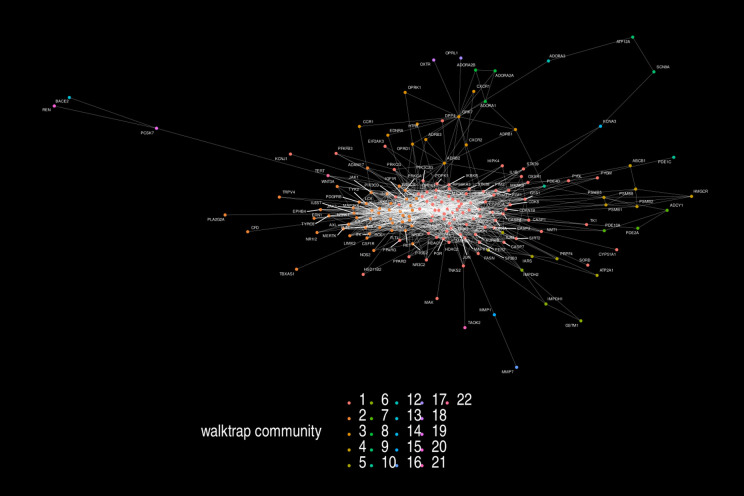
Network visualization of protein-protein interactions between targets of *G. glauca.* Nodes are colored according to the community they belong as determined by the Walktrap algorithm. A high resolution version of this image is provided in supplementary material.

We mapped the pharmacological targets of *G. glauca* using STRING-DB, revealing a network of 1,386 interactions. This network provides insights into how these targets interact within their biological contexts, positioning each protein’s role in a complex web of signaling pathways. A visualization of this network is shown in Fig 3. The full network is provided in graphml format as S3 File.

**Fig 4:**
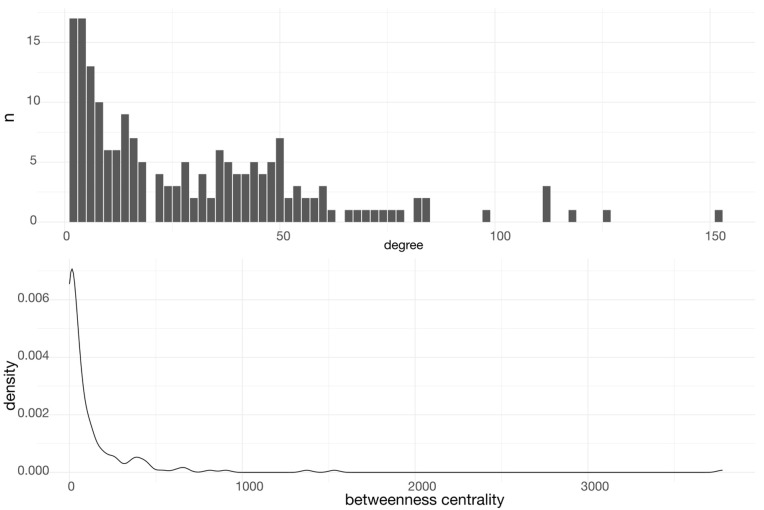
Distribution of centrality measures on the interaction network of *G. glauca* targets. Top panel shows histogram of degree centrality, while bottom panel shows density plot for betweenness centrality. In both cases, heavy-tailed distributions are shown, indicating the emergence of central elements in the network.

In Fig 4, we show the value distribution of centrality measures in this network. By the distributions, we may observe a *heavy-tailed* behavior for both degree centrality and betweenness centrality.

As such, certain proteins emerged as key players, their centrality measures underscoring their influence. Degree centrality identifies SRC, MTOR, MAPK3, and MAPK1 as the most connected proteins, each prominently involved in pathways crucial for cell growth, proliferation, and survival. Their connectivity suggests these proteins are central mediators of *G. glauca*’s pharmacological activity. Betweenness centrality, which highlights nodes that bridge different network regions, ranks SRC as the top communication hub. Other proteins with high betweenness centrality include JUN, PIK3CA, HSP90AB1, ADRB2, and MMP1, each influencing transcription regulation, signal transduction, or immune response. The whole set of network descriptors may be found in S4 File.

By looking at these individual proteins, we may gain further insight on the potential mechanisms through which *G. glauca* exhibits its known medicinal properties, as well as providing insights on possible future therapeutic applications. For instance:

- **SRC** (proto-oncogene tyrosine-protein kinase Src) supports immune processes, including proliferation, phagocytosis, and immune cell development. It advances cell cycle progression through interactions with receptors and ligands involved in macrophage-mediated inflammation, playing a recognized role in neoplastic disease treatments [4,5].
- **MTOR** (mammalian target of rapamycin) is a critical metabolic regulator, modulating growth factors, hormones, and cellular stress responses while inhibiting autophagy. MTOR-targeted therapies show promise not only in cancer but also for neurodegenerative and metabolic disorders [6,7]. Inhibiting the PI3K/Akt/MTOR pathway is an emerging therapeutic strategy in oncology [8].
- **PIK3CA** (phosphatidylinositol-4,5-bisphosphate 3-kinase catalytic subunit alpha) plays a key role in cell morphology and survival through growth factor responses. Notably, mutations in PIK3CA are linked to therapeutic resistance in about 30% of early-stage HER2+ tumors [9,10].
- **MAPK3** (Mitogen-activated protein kinase 3) is involved in processes such as cellular migration, transcription regulation, and inflammation, particularly through TNF signaling [11].
- **JUN** is a transcription factor binding to the AP1 consensus sequence, facilitating T-cell-induced cell death and steroidogenic gene expression [12].
- **HSP90AB1** (heat shock protein 90 kDa alpha, class B, member 1) functions as a molecular chaperone, essential for the regulation, maturation, and structural maintenance of target proteins. Its ATPase-dependent cycle aids in epigenetic regulation and gene expression control [13].
- **ADRB2** (beta-2 adrenergic receptor) mediates adenylate cyclase activation through epinephrine binding, with heightened affinity for norepinephrine. A classical pharmacological target, this receptor has also been studied in complex diseases such as prostate cancer therapy [14,15].
- **MMP1** (Matrix Metallopeptidase 1) exhibits catalytic activity on collagen types I, II, and III, contributing to inflammatory responses and playing a role in neuroinflammation, especially in HIV contexts [16].

The protein-protein interaction network derived from the predicted targets of *G. glauca* compounds displays a densely connected structure, with prominent nodes such as SRC, MTOR, and MAPK3 occupying central positions. These nodes correspond to molecules involved in key signaling cascades related to proliferation, immune regulation, and cellular stress. Their centrality within the network suggests that they may act as integrators of pharmacological signals arising from multiple galphimines, reinforcing the idea that the observed therapeutic properties of *G. glauca* could result from coordinated modulation of interconnected functional hubs.

### Community structure

Applying the Walktrap algorithm to the network, we identified clusters of highly connected proteins, each linked to specific biological functions. Three main communities emerged, each aligning with distinct pharmacological activities of *G. glauca*:

- **Community 1** includes proteins like MTOR, MAPK3, and JUN, which play roles in apoptosis and cell survival. This aligns with *G. glauca*’s reported antiproliferative effects.
- **Community 2** encompasses SRC, PRKCA, and HSP90AB1, central to cell growth and immune response pathways, highlighting *G. glauca*’s role in cellular regulation and immune adaptation.
- **Community 3** is associated with inflammation and immune modulation, containing targets like ADRB2 and MMP1, and supporting the traditional anti-inflammatory uses of *G. glauca*.

The community structure of the protein interaction network reveals that the predicted targets of *G. glauca* are not randomly distributed, but instead cluster into functional modules. Each of the three main communities identified by the Walktrap algorithm includes molecules associated with distinct biological processes, such as apoptosis, immune signaling, and inflammation. This modular organization supports the idea that the pharmacological effects of *G. glauca* emerge from the concerted action of its compounds on coordinated groups of targets, rather than isolated molecular events.

### Limitations and future directions

This study relies on computational prediction and network analysis to explore the pharmacological potential of *Galphimia glauca*. While these approaches provide valuable insight, they are subject to limitations. The predicted protein targets were obtained through similarity-based algorithms and have not been experimentally validated. Furthermore, network-based interpretations depend on the completeness and bias of available interaction databases, which may overrepresent well-characterized proteins. Future studies should aim to validate compound–target interactions experimentally and assess the biological effects of galphimines in cellular or organismal models. These steps will be essential to confirm the relevance of the mechanisms proposed here and to support their translational potential.

## Conclusions

This study mapped the predicted molecular targets of Galphimines A–I using a network pharmacognosy approach. The resulting structure combines a shared core of targets with peripheral, compound-specific nodes, both of which may contribute to the pharmacological effects of *G. glauca*. The analysis of protein–protein interactions, centrality patterns, and community structure suggests that these effects involve coordinated modulation of proteins associated with inflammation, immune response, and cell survival.

This work contributes new insights into the molecular mechanisms potentially underlying the activity of this traditional medicinal plant. The network-based framework presented here may also be useful for investigating the pharmacological organization of other multi-compound systems and for exploring how natural products interact with human molecular networks.

## Methods

### Selection of bioactive compounds

The pharmacologically relevant compounds from *Galphimia glauca*, known as Galphimines A-I, were extracted from the BIOFACQUIM database maintained by UNAM [17]. BIOFACQUIM is a curated repository that aggregates high-confidence compound-target associations from nine public databases. This database applies a standardized curation protocol involving duplicate removal and molecule cleaning, ensuring data reliability and coverage across various biological contexts. The database may be accessed at: https://www.difacquim.com/d-databases/

The SMILES (Simplified Molecular Input Line Entry System) strings for each galphimine were obtained from BIOFACQUIM and verified against the NCBI database (https://www.ncbi.nlm.nih.gov/). These notations allowed for consistent data entry into prediction tools for further analysis.

### Identification of pharmacological targets

To identify potential protein targets, we used SwissTargetPrediction [18], (accessible at http://www.swisstargetprediction.ch/help_mol.php). This tool predicts probable protein targets based on ligand similarity in both 2D and 3D conformations. Predictions were limited to the *Homo sapiens* species to ensure relevance for potential therapeutic applications.

The SMILES strings for each galphimine were entered into SwissTargetPrediction using default parameters, and predicted targets were ranked by probability. Targets with the highest predicted affinity were retained for network analysis, resulting in a comprehensive list of 180 unique protein targets across the nine galphimines. The top 100 highest ranked targets for each galphimine were integrated into a single bipartite network representing the pharmacological target space for *G. glauca.*

### Molecular interaction search

Protein targets identified through SwissTargetPrediction were mapped for potential interactions using the STRING database [19] (accessible at https://string-db.org/). STRING aggregates protein-protein interaction data from a variety of sources, including computational predictions, knowledge transfer from model organisms, and experimental data. Default parameters were used, with *Homo sapiens* set as the reference organism.

### Network analysis

To analyze the networks’ structures and highlight key nodes, we employed several centrality metrics:

Degree Centrality: Degree centrality was calculated as the number of direct interactions (edges) for each node (protein). Nodes with high degree centrality, such as SRC and MTOR, were flagged as potential hubs within the network.

Betweenness Centrality: Betweenness centrality was calculated using Freeman’s formula to determine each node’s importance in maintaining shortest-path connectivity across the network. This metric highlighted key bridging proteins, such as SRC and JUN.

Community Detection: We used the Walktrap algorithm to identify community structures within the network. Walktrap detects communities by simulating random walks on the network, under the principle that shorter random walks are more likely to remain within the same community. The algorithm iteratively merges nodes into communities by minimizing the mean distance between nodes within each community, creating a hierarchical structure of nested communities.

### Data accession, statistical and visualization software

All external resources were accessed in September 2024. This includes SwissTargetPrediction (http://swisstargetprediction.ch/), BIOFACQUIM (https://github.com/Daniphantom99/BIOFACQUIM), and STRING-DB (https://string-db.org/), which were used for target prediction, compound annotation, and protein–protein interaction mapping, respectively.

All network and statistical analyses were conducted in R (version 4.1.3). Network analysis was performed using the *igraph* package. Visualization of network topologies and centrality distributions were generated using *ggplot2*. Other visualizations were generated using the *gephi 0.10* software. Analysis code is available at https://github.com/MariaCambero/Analisis-Galphimia-Glauca-/

## Supporting information

S1 File SwissTargetPrediction results for Galphimines A–I. (CSV)

S2 File Node attributes from the galphimine–target bipartite network, including compound associations, centrality metrics, and Walktrap communities, graphml of the network, and high-resolution PDF of the network visualization. (ZIP)

S3 File GraphML representation of the bipartite network with node labels and metadata, accompanied by a high-resolution visualization for reference. (ZIP)

S4 File Summary of global network properties, including node and edge counts, clustering coefficient, and community structure. (TXT)

## References

[1] Sharma A, Angulo-Bejarano PI, Madariaga-Navarrete A, Oza G, Iqbal HMN, Cardoso-Taketa A, et al. Multidisciplinary investigations on *Galphimia glauca*: a Mexican medicinal plant with pharmacological potential. Mol Basel Switz. 2018 Nov 15;23(11):2985. 10.3390/molecules23112985 PMC6278297 30445751

[2] Sharma A, Cardoso-Taketa A, Choi YH, Verpoorte R, Villarreal ML. A comparison on the metabolic profiling of the Mexican anxiolytic and sedative plant Galphimia glauca four years later. J Ethnopharmacol. 2012;141(3):964–74. doi: 10.1016/j.jep.2012.03.033 22472113

[3] González-Cortazar M, Herrera-Ruiz M, Zamilpa A, Jiménez-Ferrer E, Marquina S, Alvarez L, et al. Anti-inflammatory activity and chemical profile of Galphimia glauca. Planta Med. 2014;80(1):90–6. doi: 10.1055/s-0033-1360150 24338551

[4] Byeon SE, Yi Y-S, Oh J, Yoo BC, Hong S, Cho JY. The role of Src kinase in macrophage-mediated inflammatory responses. Mediators Inflamm. 2012;2012:512926. doi: 10.1155/2012/512926 23209344 PMC3504478

[5] Roskoski R Jr. Src protein-tyrosine kinase structure, mechanism, and small molecule inhibitors. Pharmacol Res. 2015;94:9–25. doi: 10.1016/j.phrs.2015.01.003 25662515

[6] Dormond O. mTOR in human diseases. Int J Mol Sci. 2019;20(9):2351. 31083592 10.3390/ijms20092351 PMC6540159

[7] Kim YC, Guan K-L. mTOR: a pharmacologic target for autophagy regulation. J Clin Invest. 2015;125(1):25–32. doi: 10.1172/JCI73939 25654547 PMC4382265

[8] Yu L, Wei J, Liu P. Attacking the PI3K/Akt/mTOR signaling pathway for targeted therapeutic treatment in human cancer. Semin Cancer Biol. 2022;85:69–94. 34175443 10.1016/j.semcancer.2021.06.019

[9] Canaud G, Hammill AM, Adams D, Vikkula M, Keppler-Noreuil KM. A review of mechanisms of disease across PIK3CA-related disorders with vascular manifestations. Orphanet J Rare Dis. 2021;16(1):306. doi: 10.1186/s13023-021-01929-8 34238334 PMC8268514

[10] Rasti AR, Guimaraes-Young A, Datko F, Borges VF, Aisner DL, Shagisultanova E. PIK3CA mutations drive therapeutic resistance in human epidermal growth factor receptor 2-positive breast cancer. JCO Precis Oncol. 2022;6:e2100370. doi: 10.1200/PO.21.00370 35357905 PMC8984255

[11] Ronkina N, Menon MB, Schwermann J, Tiedje C, Hitti E, Kotlyarov A, et al. MAPKAP kinases MK2 and MK3 in inflammation: complex regulation of TNF biosynthesis via expression and phosphorylation of tristetraprolin. Biochem Pharmacol. 2010;80(12):1915–20. doi: 10.1016/j.bcp.2010.06.021 20599781

[12] Baumann S, Hess J, Eichhorst ST, Krueger A, Angel P, Krammer PH. An unexpected role for FosB in activation-induced cell death of T cells. Oncogene. 2003;22(9):1333–9. 12618758 10.1038/sj.onc.1206126

[13] Haase M, Fitze G. HSP90AB1: Helping the good and the bad. Gene. 2016;575(2 Pt 1):171–86. 26358502 10.1016/j.gene.2015.08.063 PMC5675009

[14] Kulik G. ADRB2-Targeting Therapies for Prostate Cancer. Cancers (Basel). 2019;11(3):358. doi: 10.3390/cancers11030358 30871232 PMC6468358

[15] Wu F-Q, Fang T, Yu L-X, Lv G-S, Lv H-W, Liang D, et al. ADRB2 signaling promotes HCC progression and sorafenib resistance by inhibiting autophagic degradation of HIF1α. J Hepatol. 2016;65(2):314–24. doi: 10.1016/j.jhep.2016.04.019 27154061

[16] Jiménez-Sousa MA, Berenguer J, Fernández-Rodríguez A, Medrano LM, Aldámiz-Echevarria T, Pérez-Latorre L. Genetic variants upstream of TNFAIP3 in the 6q23 region are associated with liver disease severity in HIV/HCV-coinfected patients: A cross-sectional study. Infect Genet Evol J Mol Epidemiol Evol Genet Infect Dis. 2019;67:112–20. 10.1016/j.meegid.2018.10.008 30336268

[17] Pilón-Jiménez BA, Saldívar-González FI, Díaz-Eufracio BI, Medina-Franco JL. BIOFACQUIM: A Mexican Compound Database of Natural Products. Biomolecules. 2019;9(1):31. doi: 10.3390/biom9010031 30658522 PMC6358837

[18] Daina A, Michielin O, Zoete V. SwissTargetPrediction: updated data and new features for efficient prediction of protein targets of small molecules. Nucleic Acids Res. 2019;47(W1):W357-64. 10.1093/nar/gkz382 PMC6602486 31106366

[19] Szklarczyk D, Kirsch R, Koutrouli M, Nastou K, Mehryary F, Hachilif R, et al. The STRING database in 2023: protein-protein association networks and functional enrichment analyses for any sequenced genome of interest. Nucleic Acids Res. 2023;51(D1):D638-46. 10.1093/nar/gkac1000 PMC9825434 36370105

