# Supplementary figures and images for: Network pharmacognosy of Galphimia glauca: Mapping the molecular landscape of a traditional Mexican medicinal plant
Source: PLoS One. 2025 Jul 1;20(7):e0317546. doi: 10.1371/journal.pone.0317546 (PMC12212526; doi:10.1371/journal.pone.0317546)

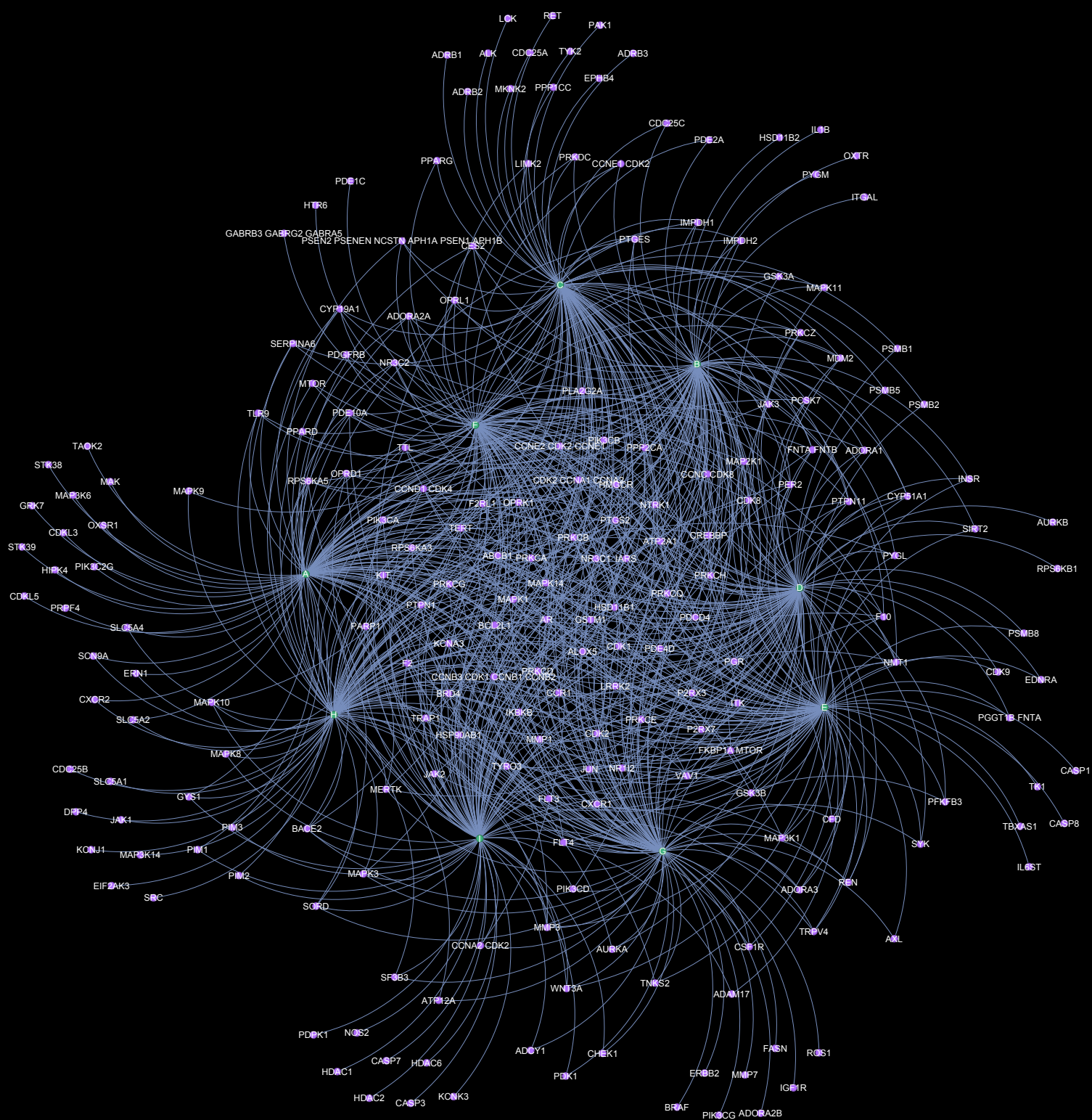

Supplement: S2 File — (ZIP) [file pone.0317546.s002.zip › F1_fullscale.pdf]
