# Supplementary material for: Network pharmacognosy of Galphimia glauca: Mapping the molecular landscape of a traditional Mexican medicinal plant
Source: PLoS One. 2025 Jul 1;20(7):e0317546. doi: 10.1371/journal.pone.0317546 (PMC12212526; doi:10.1371/journal.pone.0317546)
Supplement: S3 File — (ZIP) [file g3_annotated.pdf › g3_annotated.pdf]

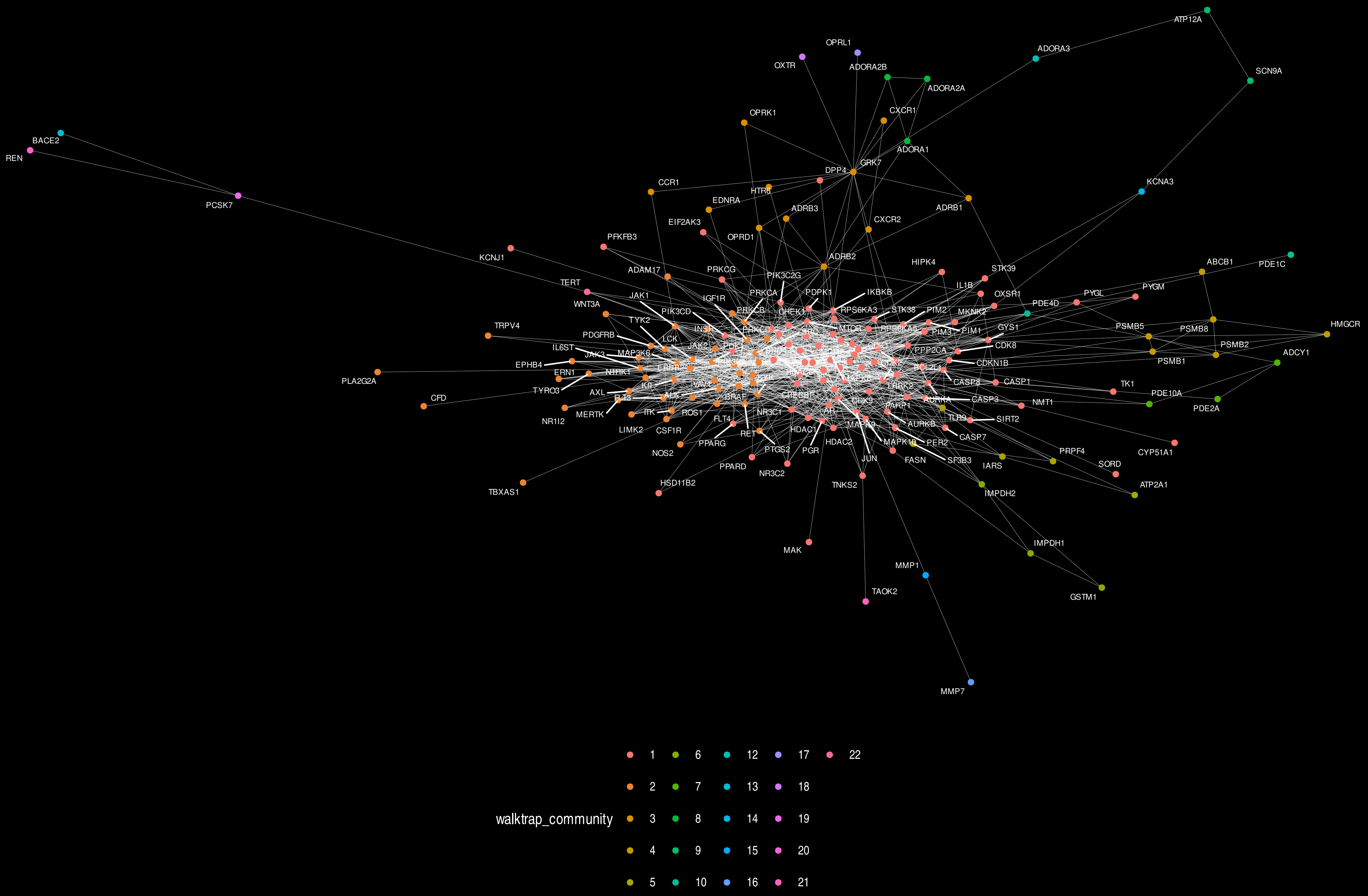
ATP12A
OPRL1
OXTR
ADORA2B
ADORA3
ADORA2A
SCN9A
CXCR1
OPRK1
ADORA1
BACE2
REN
GRK7
DPP4
PCSK7
CCR1
HTR6
KCNA3
EDNRA
ADRB3
ADRB1
EIF2AK3
CXCR2
OPRD1
PFKFB3
KCNJ1
ADRB2
HIPK4
STK39
ABCB1
PDE1C
ADAM17
PRKCG
PIK3C2G
TERT
IL1B
JAK1
PRKCA
PDPK1
IKBKB
OXSR1
PDE4D
PYGL
PYGM
WNT3A
IGF1R
PIK3CD
CHEK1
RPS6KA3
STK38
PIM2
MKNK2
TRPV4
TYK2
PRKCB
GYS1
PSMB5
PSMB8
HMGCR
PDGFRB
PIM3
PIM1
LCK
CDK8
IL6ST
JAK2
PPP2CA
PSMB2
ADCY1
JAK3
MAP3K6
PSMB1
EPHB4
CDKN1B
ERN1
NTRK1
PLA2G2A
CASP8
CASP1
TK1
TYRO3
AXL
PDE10A
CFD
FLT3
CASP3
NMT1
PDE2A
NR1I2
MERTK
ITK
ROS1
PARP1
TLR9
SIRT2
LIMK2
CSF1R
FLT4
HDAC1
AURKB
CASP7
RET
MAPK9
CYP51A1
PPARG
HDAC2
MAPK10
PER2
NOS2
PTGS2
PGR
PRPF4
SORD
PPARD
FASN
SF3B3
IARS
NR3C2
JUN
ATP2A1
HSD11B2
TNKS2
IMPDH2
TBXAS1
IMPDH1
MAK
MMP1
TAOK2
GSTM1
MMP7
walktrap_community
1
2
3
4
5
6
7
8
9
10
12
13
14
15
16
17
18
19
20
21
22
